# The Connexion between Physiology and Intellectual Philosophy

**Published:** 1843-04

**Authors:** 


Art. I.—*The Connexion between Physiology and Intellectual Philosophy*. By the Rev. John Barlow, M.A. F.R.S. F.L.S. &c. &c.—*London*, 1842. 12mo, pp. 64.

This unpretending little treatise, the substance of which was communicated to the members of the Royal Institution at one of their Friday evening meetings, contains the best general views of its subject that we have anywhere met with. The author has drawn his materials from the writings of physiologists of the highest repute; and shows himself quite *au courant* with their most recent investigations. These materials have been combined, by his own intelligent and highly-cultivated mind, into a form alike pleasing and instructive; so that this lecture may be perused with profit, as we are sure it will be with interest, by the professional as well as the general reader. The severe style of a grave philosophical treatise, the object of which is to prove rather than to illustrate the positions which the author lays down, is too apt to degenerate into a dry repulsiveness, injurious to the spread of truth, as well as to the appreciation of the labours of the writer; and we think that many an author (ourselves included) may take an instructive lesson from Mr. Barlow, as to the union of the graces of style with the solidity of true philosophy. We offer the following as examples. After pointing out the most important differences between the cerebral organization of man and that of the highest among brutes, he thus proceeds to illustrate the superiority of his governing *will*, to the instinctive impulses which he shares with the lower animals:

"The first instinctive impulse is to preserve life. Look at a wrecked vessel! There is one man there, ordering and directing all on board: the only remaining boat is lowered; he is careful to see it filled with the persons crowded about him, it pushes off, and where is he? He is there on the deck of that sinking ship; the boat would not hold *all*, and he has refused a place in it, and remained to perish, rather than sacrifice one life committed to his charge. He knows that death awaits him: he has been urged to save himself: and yet he is there! What is the impulse which prompts him thus to contravene the first great law of animated nature?

"Sleep, again, is among our most imperious needs, for the want of it gradually destroys life. There lies a sick man in his bed, senseless, in the last stage of an infectious fever: and there is one watching beside him, looking pale and exhausted, but who sleeps not, stirs not, though her young life is wasting away with fatigue, and exposed to contagion: and she knows it, and has calculated that the same grave will receive both. What nerve of all that fine machinery has impelled her to this course?

"Look at the astronomer in his observatory! The night is far advanced and he is chilled and fatigued; yet he remains with his eye at the telescope—for what? To carry on a series of observations, which perhaps in two generations more may give as its result the knowledge of some great law of the material universe: but he will be in his grave long ere he can expect that it will be ascertained. He sits down to his calculations, and he forgets his meals, sees nothing, hears nothing, till his problem is solved! No sense prompts him to this sacrifice of rest and comfort. But do we call these persons insane? No—we honour them as the excellent of the earth; admire their lives, and wish that, when the occasion comes, we may have courage so to die.

"I know of but one solution of the difficulty; there must be some element in man which we have not yet taken account of; some untiring, undying energy, which eludes, indeed, the fingers and the microscope of the anatomist, but which exercises a despotic sway over the animal mechanism, and takes possession of it for its own use, to the point of exhausting and finally destroying it.

. . . . . . . .

"If we look through nature, we shall find that the happiness of the organized being consists in the accomplishment of its end of existence. Animals while supplied with food and propagating their kind, are happy: their span of life is long enough for all the enjoyments they require; but man's life is insufficient for his wishes, and these gross pleasures disgust and weary him. Where is *his* happiness then? We have seen it! The captain of the wrecked vessel feels his heart swell with proud delight, as he awaits death with a consciousness of having done what, if he were an animal only, would be an act of the wildest insanity. The fair girl, before whom all the pleasures of life were smiling, despises them, and finds her joy iu dying with the object of her affections, because she *feels*, even if she does not argue, that thus they will still be united. The astronomer has no greater delight than to pursue knowledge which affords him neither fame nor profit; though it be only to be gained at the expense of fatigue at any rate, and probably of health.........We have seen that all animated nature seeks the end of its being, and is happy in attaining it: if man then be akin to that Ruling Will, which both he and the universe own as Lord, the ultimate object of his existence must be alike happiness; and we can figure none to ourselves for such a being, but pure benevolence and perfect knowledge."

Illustrations, such as these, convey to most minds a stronger conception of a great principle, than the didactic enunciation of it, however clearly expressed. Those who heard the clinical lectures of Sir C. Bell, will remember how felicitously they were often introduced by him. Yet there may be danger of running into an extreme, and of making the real object subservient to an attempt at rhetorical display. Our readers will be best able to understand our idea of a good lecture upon an abstract subject, by perusing the one from which we have quoted these specimens.

